# Rapid Detection of Wheat Ears in Orthophotos From Unmanned Aerial Vehicles in Fields Based on YOLOX

**DOI:** 10.3389/fpls.2022.851245

**Published:** 2022-04-27

**Authors:** Yao Zhaosheng, Liu Tao, Yang Tianle, Ju Chengxin, Sun Chengming

**Affiliations:** ^1^Jiangsu Key Laboratory of Crop Genetics and Physiology/Jiangsu Key Laboratory of Crop Cultivation and Physiology, Agricultural College of Yangzhou University, Yangzhou, China; ^2^Jiangsu Co-Innovation Center for Modern Production Technology of Grain Crops, Yangzhou University, Yangzhou, China

**Keywords:** small target, spike, YOLOX, UAV, Orthophoto, BiFPN

## Abstract

Wheat ears in unmanned aerial vehicles (UAV) orthophotos are characterized by occlusion, small targets, dense distribution, and complex backgrounds. Rapid identification of wheat ears in UAV orthophotos in a field environment is critical for wheat yield prediction. Three improvements were achieved based on YOLOX-m: mosaic optimized, using BiFPN structure, and attention mechanism, then ablation experiments were performed to verify the effect of each improvement. Three scene datasets were established: images were acquired during three different growing periods, at three planting densities, and under three scenarios of UAV flight heights. In ablation experiments, three improvements had increased recognition accuracies on the experimental dataset. Compared the accuracy of the standard model with our improved model on three scene datasets. Our improved model during three different periods, at three planting densities, and under three scenarios of the UAV flight height, obtaining 88.03%, 87.59%, and 87.93% accuracies, which were, respectively, 2.54%, 1.89%, and 2.15% better than the original model. The results of this study showed that the improved YOLOX-m model can achieve UAV orthophoto wheat recognition under different practical scenarios in large fields, and that the best combination were obtained images from the wheat milk stage, low planting density, and low flight altitude.

## Introduction

Wheat yield is calculated from the number of spikes per unit area, the number of grains per spike, and the weight of grains. In agricultural production, especially in wheat cultivation and breeding, determination of the number of spikes per unit area still relies on manual work, which introduces human error during prolonged, intensive work. Therefore, a fast, accurate method for counting wheat spikes in a large field environment is essential.

Compared with the time-consuming and laborious manual counting, modern information tools such as machine-learning methods, image analysis techniques, and artificial intelligence technologies can significantly improve the efficiency of wheat spike counting (Xu et al., 2020). Research on wheat-ear recognition has developed considerably in the last decade and can be broadly classified into traditional image processing methods, deep-learning network methods, and other methods. Using first-order and high-order methods, Frdric et al. (2012) attempted to extract wheat-ear texture features and used the K-means algorithm for ear segmentation and wheat-ear counting by setting thresholds. Cointault and Gouton (2007) combined color features based on texture features for wheat-ear segmentation and used the skeleton method to solve the problem of overlapping wheat ears during counting to improve the accuracy of wheat-ear recognition. Tao et al. (2014) used image processing techniques to complete the segmentation of wheat ears in a large field, and added the corner-point detection method to achieve automatic counting of wheat ears. Fernandez-Gallego et al. (2020) utilized a drone to collect images of wheat fields and compared the effectiveness of eight machine learning methods on wheat-ear recognition. Their results showed that the random forest method had the highest accuracy.

Owing to broad applicability and high accuracy, deep-learning methods have become a new means to address the challenges encountered in wheat counting. Sadeghi-Tehran et al. (2019) constructed wheat feature models and fed the models into convolutional neural networks to achieve semantic segmentation and automatic counting of wheat. In addition, TasselNetv2 (Xiong et al., 2019), mobileNetV2 (Khaki et al., 2021), YOLOV4 (Yang et al., 2021), EfficientDet (Wang et al., 2021), LPNet (Misra et al., 2020), and other deep-learning networks have shown advantages in wheat counting.

The challenges to effective wheat detection have promoted the rapid development of machine learning algorithms in wheat-head detection. Many wheat datasets have emerged, among which the most popular one is the Global Wheat Head Detection (GWHD) dataset (David et al., 2021). These developments have played a significant role in advancing wheat-detection algorithms. The equipment used to acquire wheat pictures in this dataset was a digital camera, shooting from a height between 1.8 and 3 m above the ground and a ground sampling distance (GSD) of 0.10–0.62 mm/px.

Relative size is mainly used to define small targets in target detection. The relative size is defined according to the Society of Photo-Optical Instrumentation Engineers (SPIE), and a small target is defined as a target area of fewer than 80 pixels in a 256 × 256 image, i.e., less than 0.12% of 256 × 256 is a small target (Yang et al., 2016). Most current research vehicles for wheat-ear detection are far beyond the small target range: high-definition images are obtained by high-definition cameras hand-held or mounted on a shelf and photographed at a closer distance, which makes the features of wheat ears clear and easy to be extracted to more features by deep networks, and the recognition accuracy can reach 98% (Zhou et al., 2018; Xiong et al., 2019; Li et al., 2021). However, this method does not apply to actual production practices and does not achieve field-wide or larger-scale wheat-ear detection. After wheat heading completed, clear images need to be captured using unmanned aerial vehicles (UAV) and using orthophoto stitching technology, then detect wheat ears.

The ground sampling distance of wheat-ear images obtained by UAV is greatly affected by the flight height of the UAV. Taking DJI Inspire2 as an example, the experiment showed that after wheat head was presented, visible, and fully emerged. The UAV flight height was below 10 m, the wind generated by the propeller blew the ears of wheat about, making them shake and thus affecting the clarity of shooting and multi-photograph synthesis of orthophoto images. Flying the UAV too high made it impossible to extract wheat-ear features by a deep-learning network, yielding poor results. Therefore, a better method was needed for large-scale UAV orthophoto detection of small wheat-ear scenes with low GSD, high density, and small targets.

YOLOX is a series of YOLO improvement algorithms introduced by Kuang-Shi Technology (MEGVII) in 2021 (Ge et al., 2021). YOLOX provides the following improvements over YOLOv3: (1) decoupled head (by decoupling the prediction branches, the convergence speed improves, as does AP by 4.2%, over the non-decoupled end-to-end method); (2) data augmentation (using Mosaic and Mixup and turning off data augmentation for the last 15 epochs to prevent excessive data augmentation); and (3) anchor improvement (using Anchor-free, improving multi-positives and SimOTA, reducing training time, and improving prediction accuracy). This is a good new model. Currently it is not used much in articles. Panboonyuen et al. (2021) utilizing pre-training Vision Transformer (ViT) as a backbone, apply Feature Pyramid Network (FPN) decoder detection of Road Assets, It significantly outperforms other state-of-the-art (SOTA) detectors. Zhang et al. (2021) used the YOLOX algorithm to detect vehicle targets in UAV images, and through a self-made dataset, the detection results surpassed traditional algorithms. At present, there is no article using YOLOX to detect wheat ears.

In this paper, we propose a method to obtain large-scale orthophotos of wheat fields using UAVs with telephoto lenses. We validate the algorithm for wheat-spike detection performance phenotypes under three periods, three densities, and three flight height scenarios using the improved You Only Look Once (YOLOX) deep-learning network algorithm. The improved YOLOX-m model achieves a good effect on low-resolution images. Realize the identification of dense small target wheat ears in large size (1,280pixels × 1,280pixels) images, it’s favorable to the identification of wheat ears in large field orthophotos. And the best UAV orthophoto recognition is obtained from the wheat milky stage, low planting density, and low flight altitude. The improved YOLOX model exhibits the higher classification accuracy and the different scene adaptation capability.

## Materials and Methods

### Experimental Designs

**Figure 1:**
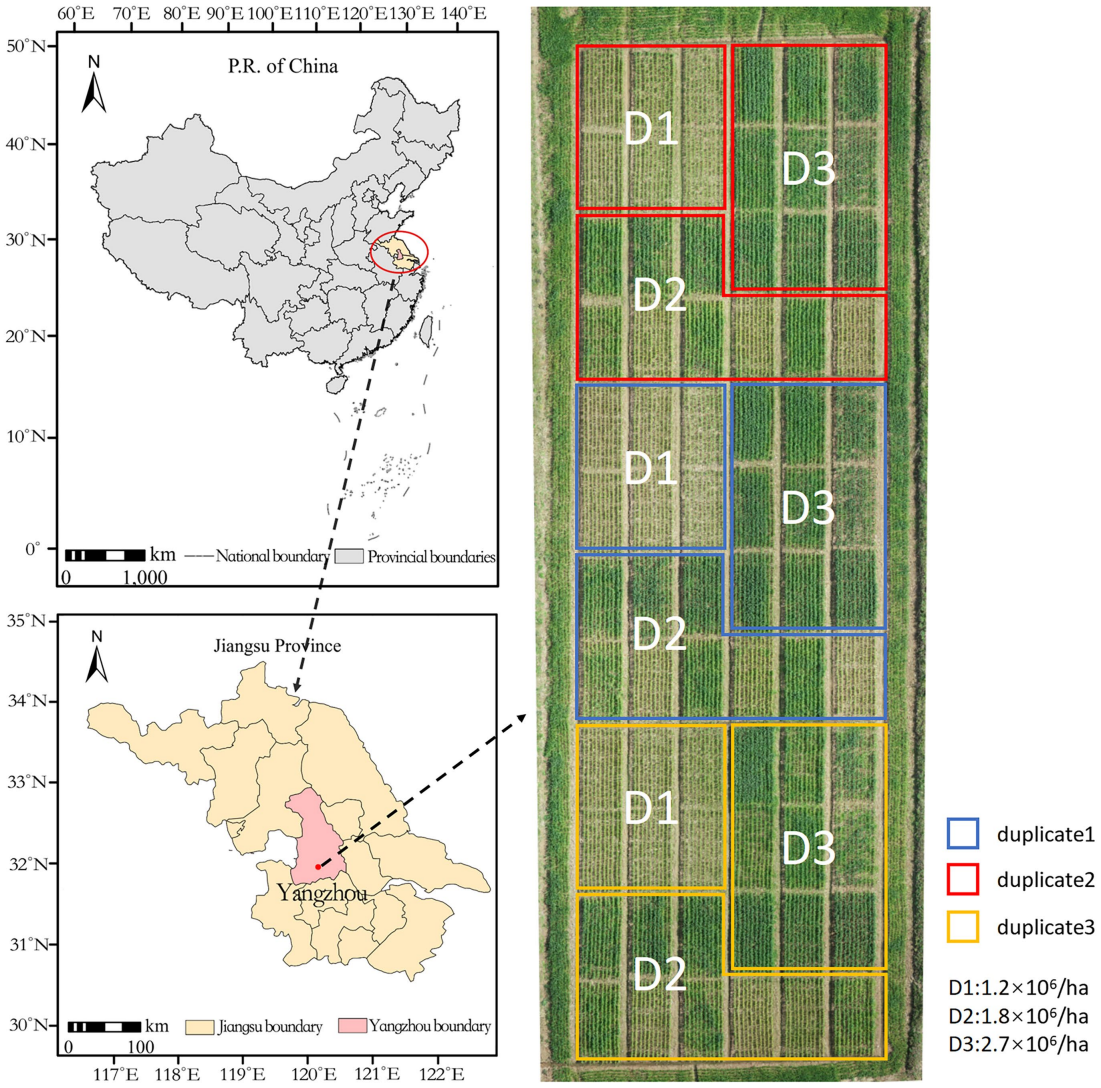
Distribution of test sites and test fields.

This study was conducted at the Fengling Experimental Base of Yangzhou University in Yangzhou City, Jiangsu Province, China (32° 30′ 7″, 119° 13′ 54″) using a 75 m × 25 m field size, with each plot measuring approximately 20 m^2^. Wheat plant conformation and spike morphology are influenced by variety. Yangmai 23, which has a large planting area, was selected as the experimental variety, with three densities: 1.2 × 10^6^/ha (D1), 1.8 × 10^6^/ha (D2), and 2.7 × 10^6^/ha (D3), and replicated three times (Figure 1). The sowing date was October 11, 2020, the planting method was mechanical strip sowing, fertilization was consistent, and other cultivation measures were consistent with local customs.

### Image Acquisition

**Figure 2:**
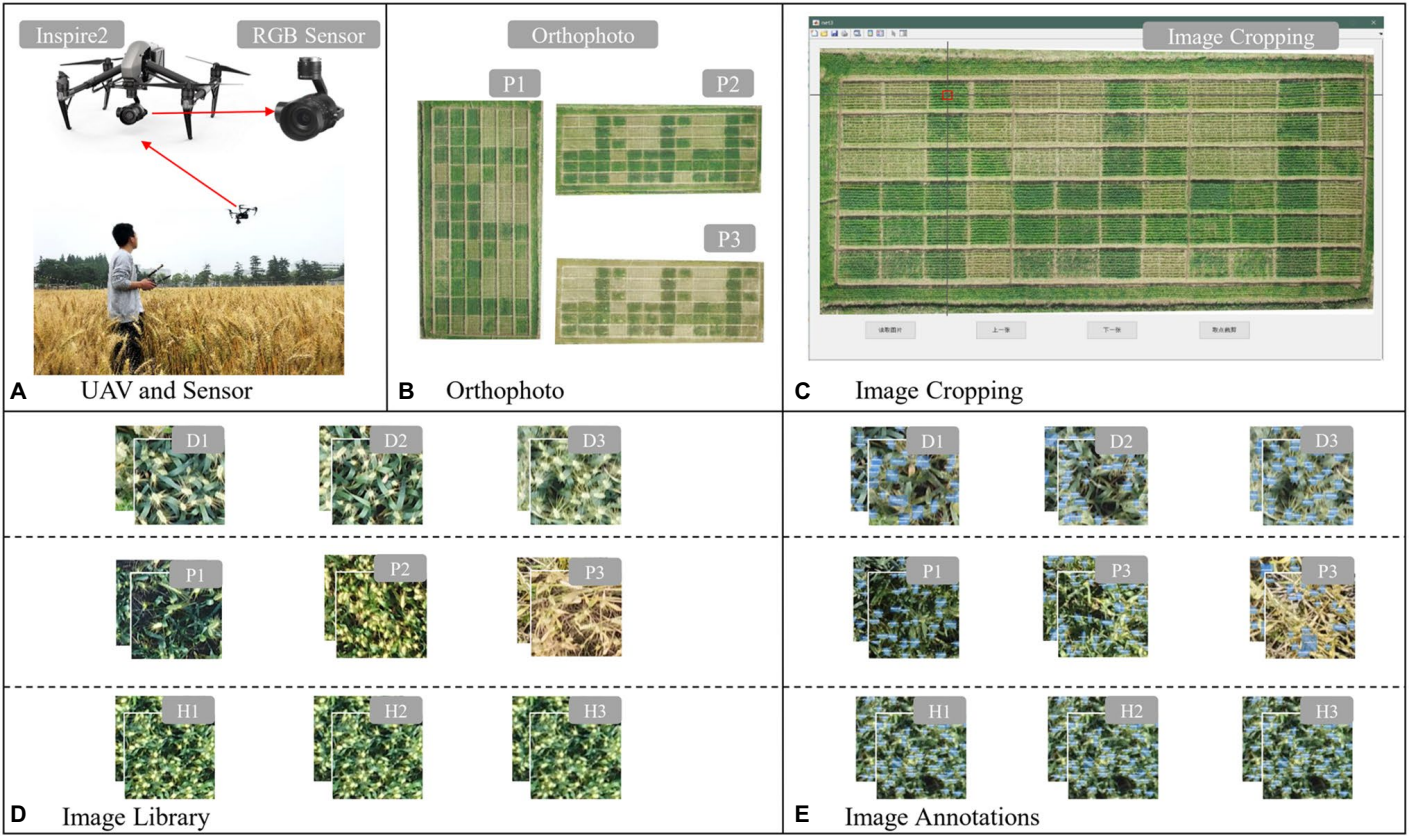
Image acquisition and dataset creation methods: **(A)** unmanned aerial vehicle (UAV) and sensor, **(B)** orthophoto, **(C)** image cropping, **(D)** image library, and **(E)** image annotations.

This study used a DJI insprire2 (inspire2, from SZ DJI Technology Co. Ltd., Shenzhen, China) multi-rotor UAV equipped with a Zenmuse X5S (SZ DJI Technology Co. Ltd., Shenzhen, China) camera, an Olympus M. Zuiko 45 mm/1.8 (Olympus Co. Ltd., Tokyo, Japan) lens (Figure 2A), and DJI GS Pro (SZ DJI Technology Co. Ltd., Shenzhen, China) ground station software to conduct one UAV mission at the flowering (P1), milking (P2), and maturity (P3) stages of wheat. The flight parameters were set at 78% heading overlap, 80% bypass overlap, the flight height at 20 m (H1), 25 m (H2), and 30 m (H3). The orthophoto reconstruction of the acquired flight data was performed using DJI Terra (SZ DJI Technology Co. Ltd., Shenzhen, China), and orthophotos were exported for the next step (Figure 2B).

### Scene Dataset Production

According to the experimental design, three wheat scenes were divided into different periods of wheat scenes (P series), different densities of wheat scenes (D series), and different resolutions of wheat scenes (H series):

P series: D2 image region orthophotos of three periods from P1 to P3 were selected as the image source,

D series: P2 period image orthophotos under three density treatments from D1 to D3 are selected as the image source,

H series: P2 period image orthophotos of three flight heights from H1 to H3 are selected as the image source.

To improve the efficiency of image cropping, a software “Crop Assistant” (Figure 2C) was developed to quickly crop the image to a specified size. The mouse is used to let the cross-auxiliary line move to the image area to be cropped, followed by clicking at the center point. The image size of 200 × 200 pixels can be intercepted with the cross as the center point, and the image is automatically named and saved to a preselected folder according to the rules (Figure 2D). The user then manually labels the cropped images with wheat ears and generates the corresponding xml file, which contains information such as image size, label name, and target location (Figure 2E). The number of statistical images ( $N_{img}$ ) and the total number of labeled boxes ( $N_{lab}$ ) are shown in Table 1. There were 3,171 images labeled in three series of nine-scene datasets, with a total number of wheat ears of 82,873. The amount of data met the number of datasets required by a deep-learning network. Each dataset is randomly divided into a training set, a validation set, and a test set in a 7:2:1 ratio for model training.

**Table 1:** Dataset base information.

Dataset	P Series			D Series			H Series			Total
	P1	P2	P3	D1	D2	D3	H1	H2	H3	
$N_{img}$	370	395	390	368	355	350	302	327	314	3,171
$N_{lab}$	8,547	9,717	9,321	7,544	8,591	10,640	7,399	9,810	11,304	82,873

$N_{img}$ , *total number of images and* $N_{lab}$ , *total number of label boxes*.

## YOLOX-m Model Improvement and Evaluation Metrics

YOLOX has a good recognition effect on target in the existing model, but there is still room for improvement. Sun et al. (2021) compared the performance of YOLOX and Deformable DETR (Deformable transformers for end-to-end object detection) models in the identification of bok choy seedlings from Weeds. The results showed that YOLOX was the optimal model, and got better average precision and identification speed. In order to better apply YOLOX to wheat ear detection, we have made three improvements: (1) data augment: optimized mosaic, added image random brightness processing, and limited the scaling ratio to 1–3. (2) Added a channel attention mechanism in backbone. Extract information that is more important to the task objective from numerous feature information. The efficiency and accuracy of model processing can be improved. The channel attention mechanism has been proven to use more attention resources to acquire high-value information and compress useless information (Woo et al., 2018). (3) The neck adopts the BiFPN (Bi-directional Convolutional Block Attention Module) structure. It uses learnable weights to learn the importance of different input features, repeatedly applying top-down and bottom-up multi-scale feature fusion (Tan et al., 2020).

**Figure 3:**
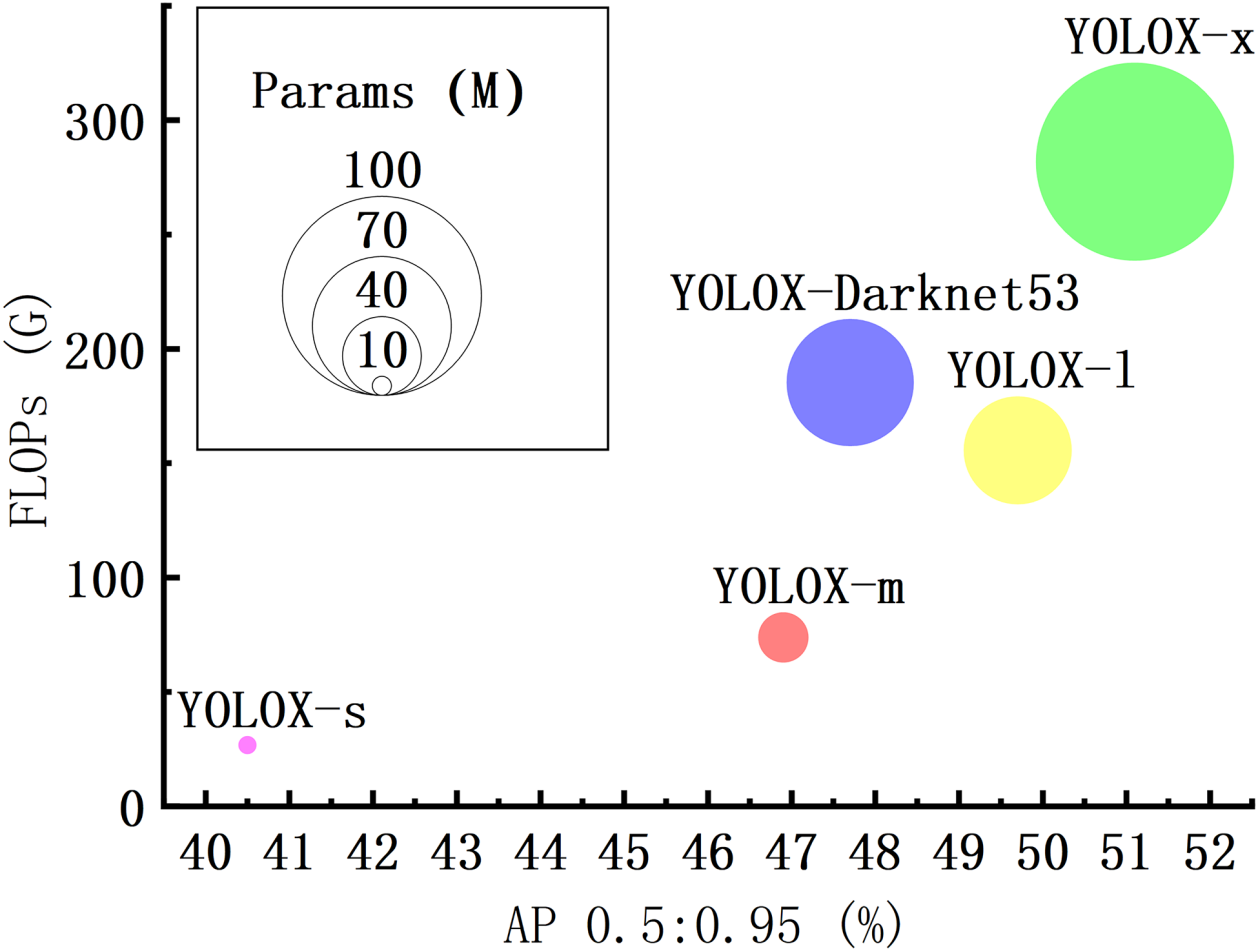
Accuracy, number of parameters, and computational overhead of each YOLOX model.

The YOLOX series includes YOLOX-s, YOLOX-m, YOLOX-l, YOLO-x and YOLOX-Darknet53, the model size (parameters) and accuracy (usually expressed in mean average precision, mAP) increase in turn under the same conditions. Larger size model, greater arithmetic power required. Floating point operations (FLOPs) can be used to measure the complexity of the model, the larger FLOPs need more arithmetic power. The mAP (0.5) of YOLOX-m is 15.80% higher than that of YOLOX-s. YOLOX-l, YOLOX-x and YOLOX-Darknet53’ mAP are only 5.97%, 8.96%, and 1.71% higher than YOLOX-m, respectively, but the FLOPs and the parameters of them are much higher than YOLOX-m (Figure 3). Considering the task scenario and hardware requirements, combined with the determination of YOLOX accuracy, the number of parameters, and arithmetic power of each model, YOLOX-m has high prediction accuracy, small parameters, and low computational overhead compared with other YOLOX series model. Finally, we selected the YOLOX-m model for optimization and testing.

**Figure 4:**
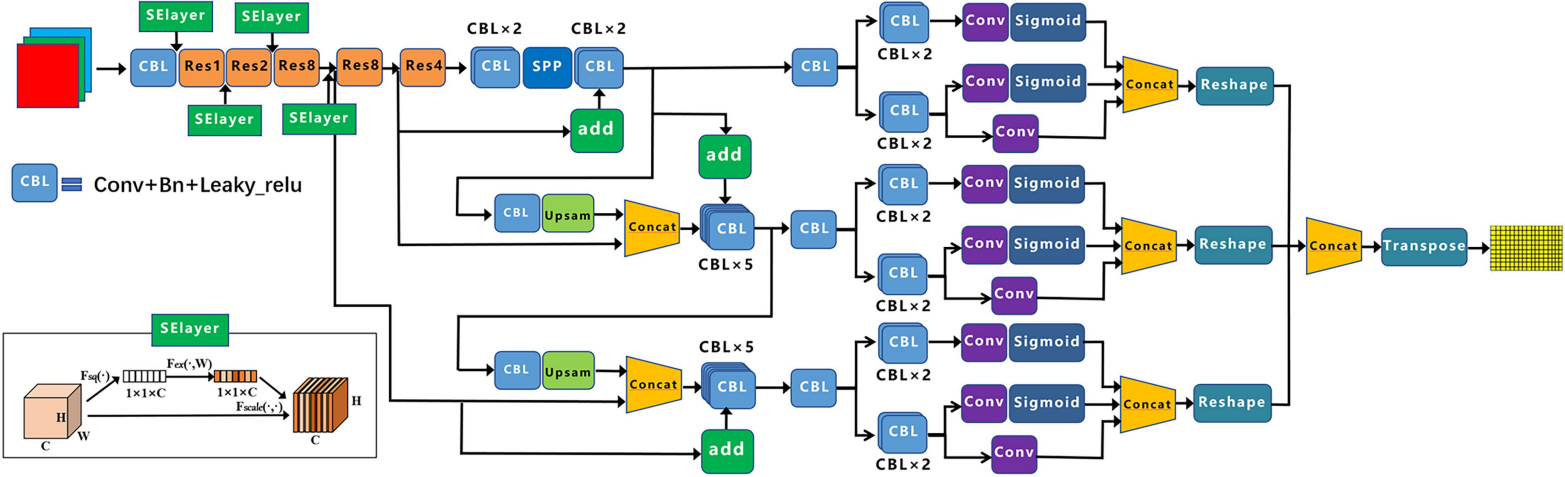
Improved YOLOX-m framework diagram.

Based on YOLOX-m, this paper proposes the following improvements for low-resolution, dense target scenes (the improved YOLOX-m network framework is shown in Figure 4).

### Data Augmentation and Mosaic Optimization

**Figure 5:**
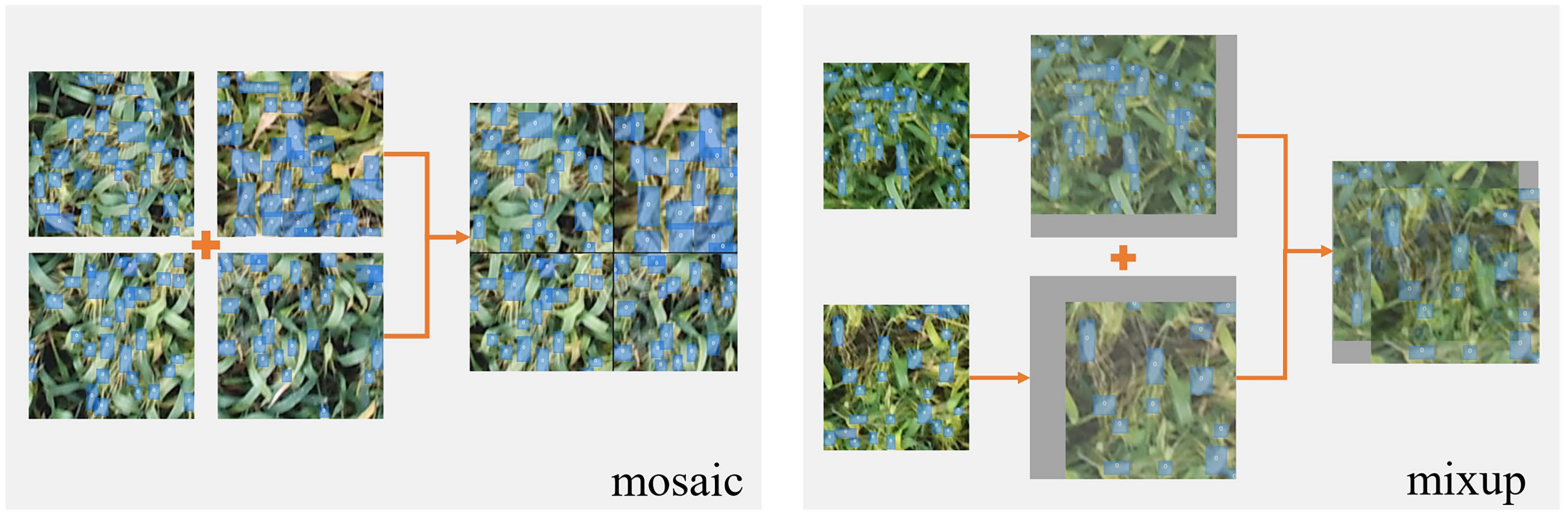
Schematic diagram of mosaic and mixup processing.

This paper describes a modified mosaic method for data augmentation and expansion. A mosaic is four images stitched together into a new image after random changes, such as flipping and scaling while processing the labels corresponding to the target objects (Yun et al., 2019; Figure 5). Experiments have shown that mosaic enhancement in model training is easier to detect in small targets, such as wheat ears (Kisantal et al., 2019). Considering the low resolution of our dataset and the small, dense nature of wheat targets, a light random variation code and a restricted mosaic scaling index were added to improve the network learning. We made the following improvements to the algorithm: (1) by converting RGB to HSV, setting the boosted V-segment value, and then converting the result to RGB, we changed the image’s brightness to simulate random changes in lighting; and (2) we limited the scale parameter for the mosaic in 1:3, i.e., instead of shrinking the image, we randomly zoomed-in up to 3x. For low-resolution and small target objects, the input network improved considerably over the original image after zooming-in.

In addition, the mixup algorithm was also used, which first read an image to scale up to a 640 × 640 image while calculating the scaled annotation frame. Next, a randomly selected image was also filled and scaled to 640 × 640 pix, and the scaled label box was calculated. The fusion factor was set, and the two images after the change were weighted and fused to finally obtain a mixup image (Zhang et al., 2017), where the annotation frames of the two images exist superimposed.

### Backbone Added the Channel Attention Mechanism

The innovation of the Squeeze-and-Excitation Layer (SElayer) network focuses on the relationship between channels, with the aim for the model to automatically learn the importance of different channel features (Hu et al., 2018). In essence, convolution is the fusion of features over a local region, including the spatial (H and W dimensions) and inter-channel (C dimension) fusion of features. Small targets have weak feature representation on their own, and detection in which more feature information needs to be learned requires deepening the network structure. The channel attention mechanism allows the neural network to focus on the channels more relevant to the target task and achieve a reasonable weight distribution. Extract information that is more important to the task objective from numerous feature information, it can improve the efficiency and accuracy of model processing. The channel attention mechanism has been proven to use more attention resources to obtain high-value information and compress useless information.

Figure 4 shows the SElayer structure, which uses global average pooling. The feature map of c channels, H × W, is compressed into C channels, 1 × 1, by Equation (7); 1 × 1 × C contains global spatial information and is compressed into a channel descriptor (channel descriptor).


(1) $$F_{sq}\left(u_{c}\right)=\frac{1}{H\times W}\overset{H}{\underset{i=1}{\sum }}\;\overset{W}{\underset{j=1}{\sum }}u_{c}\left(i,j\right)$$

The result of the global pooling of squeezed channels (which can be considered a C-dimensional vector) is fully connected to obtain a C/r-dimensional vector, which Relu activates. It is then fully connected again to change the C/r-dimensional vector back to a C-dimensional vector and is finally activated by a sigmoid function so that values lie between 0 and 1. This is the obtained weight matrix.


(2) $$F_{ex}\left(z,W\right)=\sigma \left(g\left(z,W\right)\right)=\sigma \left(W_{2}\delta \left(W_{1}z\right)\right)$$

The SElayer is added to layers 1, 2, 3, and 4 after Conv + Bn + Leaky_relu (CBL). The SE module is designed mainly to improve the model’s sensitivity to channel features. The module is lightweight and can be applied to existing network structures to improve performance, with only a small increase in computation.

### The Neck Adopts the BiFPN Structure

YOLOX uses YOLOv3 with added SPP components as the benchmark network and Neck as the FPN structure. We added the BiFPN structure to the network, as shown in Figure 4. The target detection task for small objects is difficult because large objects occupy many pixel points, but small objects have few. In the convolution process, as the convolution goes deeper, the features of large objects are readily retained, while the features of small objects are easily ignored after multiple convolutions. Therefore, the FPN structure is generated, which fuses the detailed information of the lower layers and the semantic information of the higher layers, thus increasing the perceptual field of the lower layers and enabling the lower layers to obtain more contextual information when performing small object detection (Tan et al., 2020). BiFPN is a weighted bi-directional feature pyramid network that allows fast, straightforward multi-scale feature fusion to pursue a more efficient multi-scale fusion.

### Evaluation of the Model Performance

The validation set in the respective dataset is used as a reference to evaluate the accuracy of the model prediction. The following metrics are selected in this paper to measure the accuracy of the model.

#### IOU Loss

In the IOU evaluation criteria, the L1 loss and L2 loss are obtained by summing the four coordinates of the bounding box after finding the losses separately, which ignores any correlation between the coordinates. However, the evaluation does need to consider the correlation between the coordinates. The calculation formula is as follows:


(3) $$U\;loss=-\ln\frac{Intersection\left(Prediction,Groundtruth\right)}{Union\left(Prediction,Groundtruth\right)}$$

where Ground truth is the true frame consisting of ${\tilde{x}}_{t},{\tilde{x}}_{b},{\tilde{x}}_{l},\mathrm{and}\;{\tilde{x}}_{r}$ , and Prediction is the prediction frame consisting of $x_{t},x_{b},x_{l},\mathrm{and}\;x_{r}$ . The IOU loss is obtained by evaluating −ln(IOU) after determining the IOU. Relative to L2 loss, the IOU loss increases with the number of iterations with lower loss, and the prediction frame is more accurate (Yu et al., 2016).

#### Average Precision (AP50)

To assess the accuracy of the network, we tested AP50. AP50 is the average precision when the IOU of the prediction frame and that of the real frame are greater than 0.5. A higher AP means that the accuracy of the network is higher. The formula for AP is as follows:


(4) $$AP=\overset{N}{\underset{k=1}{\sum }}\underset{k\geq k}{\max P}\left(\tilde{k}\right)\Delta Re\left(k\right)$$

where *P* is the accuracy rate (Equation 5), and Re is the recall rate (Equation 6).


(5) $$P=\frac{TP}{TP+FP}\times 100\%$$

(6) Re=TPTP+FN×100%

True positive (TP) represents that the samples are predicted correctly and are actually positive. False-positive (FP) represents that the samples are predicted to be positive but are actually negative. In addition, False-negative (FN) represents that the samples are predicted to be negative but are actually positive.

#### Frame Per Second

The number of frames per second (FPS) is an important indicator to examine the real-time performance of the model. An adequate FPS can meet the demand in practical applications.

#### RMSE and *R*^2^

In addition, metrics such as root mean square error (RMSE) and coefficient of determination (*R*^2^) are used to evaluate the wheat head counting performances. The lower RMSE and higher *R*^2^, the better performance of the model. Their counting equations are as follows:


(7) $$RMSE=\sqrt{\frac{1}{n}\overset{n}{\underset{k=1}{\sum }}{\left(Truth_{k}-Predicted_{k}\right)}^{2}}$$

(8) $$R^{2}=1-\frac{\sum_{=1}^{n}{\left(Truth_{k}-Predicted_{k}\right)}^{2}}{\sum_{k=1}^{n}{\left(Truth_{k}-Truth_{k}\right)}^{2}}$$

For the improved model, we conducted ablation tests and performed statistical analysis on three series of datasets. Performance tests were conducted using the improved YOLOX-m model for different periods of wheat-ear scenes (P1–P3), different density wheat-ear scenes (D1–D3), and different resolution scenes (H1–H3).

## Results and Analysis

### Ablation Experiments With Improved YOLOX-m Model

We randomly selected 350 annotated datasets from all annotated images for the mixed dataset test and divided the training and validation sets in the ratio of 8:2. Using the original YOLOX version of YOLOX-m as the baseline, we tested three optimization schemes: data augmentation to improve mosaic, adding SElayer, and using BiFPN. The platform configuration used for the ablation experiments used the Intel(R) Xeon(R) CPU E7-8880 v4 (2.20Ghz) × 4, RAM: 256 GB, GPU: Quadro RTX 5000 with 16 GB of video memory, CUDA version 10.0, and cudnn version 7.4. Other experiments in the following are also based on this platform. The training epoch for all models was 300 iterations, and the batch size was 6.

**Figure 6:**
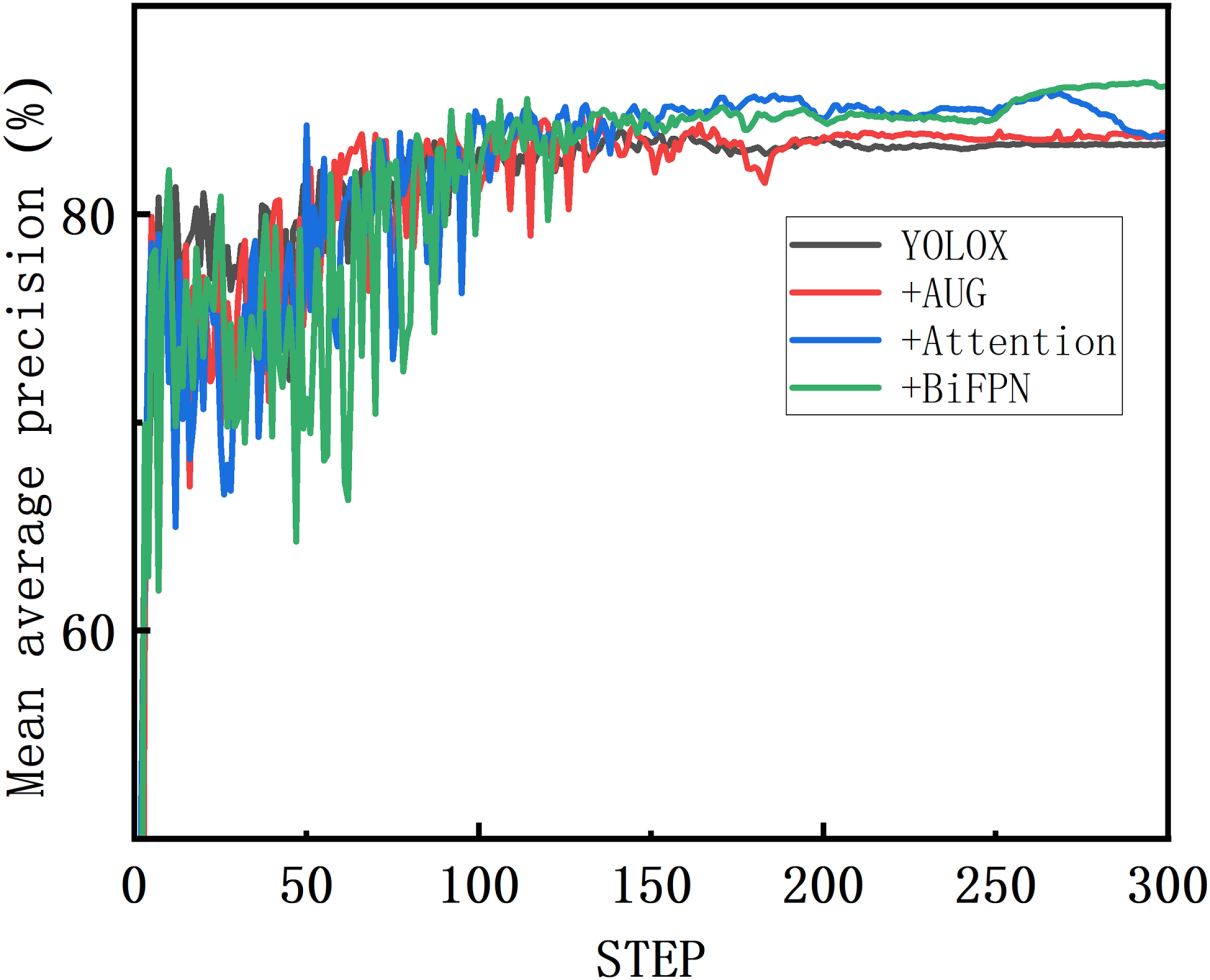
Ablation test of YOLOX-m improved model on mixed dataset.

Compared with the standard YOLOX-m, the improved YOLOX-m-based method had the highest accuracy with an AP50 of 86.34% (Table 2), which was 2.74% higher than that of the standard YOLOX-m, and a speed of 40.16 FPS, which could achieve the task of wheat spike detection accurately. The standard YOLOX-m reached a high point and converged faster in the early stage. The model emerged with a larger fluctuation early after the data enhancement optimization was turned on. The fluctuation enhanced sequentially after the Attention and BiFPN were turned on, and both gradually converged after the 150th epoch (Figure 6). Finally, the models with standard YOLOX-m and data-enhanced optimization enabled maintained a flat trend until the end of the training. In contrast, the model with Attention enabled showed an upward change and then a downward change after the 250th epoch, and the model with BiFPN enabled showed a continuous upward trend after the 250th epoch.

**Table 2:** Accuracy and performance of ablation experiments with the improved YOLOX-m model.

Model improvement	AP50 (%)	FPS
YOLOX-m	84.04	39.86
+AUG	84.69 (**+0.65**)	40.37
+Attention	85.89 (**+1.20**)	40.53
+BiFPN	86.34 (**+0.45**)	40.16

*The bold values means the difference between this value and the previous value*.

### Performance of the Improved Model on Different Scenario Datasets

**Figure 7:**
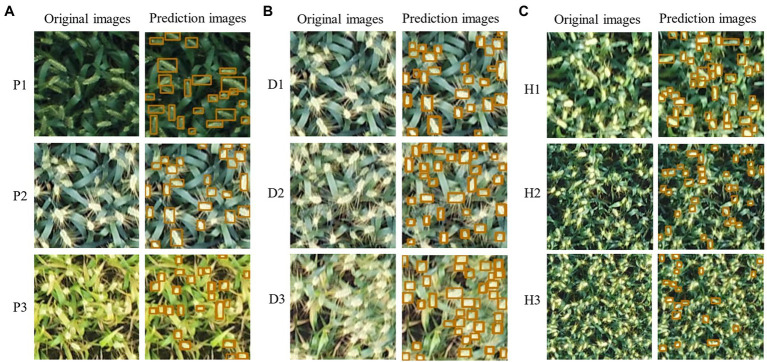
Prediction effect of the improved model on the three series datasets: **(A)** P series, **(B)** D series, and **(C)** H series.

The training results of the improved model on three series of datasets are shown in Figure 7. Comparing the test results on the original YOLOX-m network for a total of nine datasets in three series, the accuracy (AP50) of the improved model were all improved to different degrees (Table 3). The highest increase on the D2 dataset, which increased by 3.1%. P2 dataset increase 2.54% and the lowest increase on the H3 dataset, which increased by 0.78%. The improved YOLOX-m model can increase wheat recognition accuracy of UAV orthophotos under different practical scenarios in large fields. The following is a detailed analysis of three scenarios:

**Table 3:** Comparison of accuracy and IOU loss between original and improved networks.

Dataset	Origin model		Improved model	
	AP50 (%)	IOU loss	AP50 (%)	IOU loss
P1	78.58	2.25	80.70 (+2.12)	1.18
P2	85.49	2.25	88.03 (+2.54)	1.24
P3	75.93	2.31	77.79 (+1.86)	1.33
D1	85.70	2.29	87.59 (+1.89)	1.42
D2	81.83	2.33	84.94 (+3.11)	1.59
D3	73.51	2.68	76.23 (+2.72)	1.26
H1	85.78	2.23	87.93 (+2.15)	1.28
H2	72.16	2.36	74.35 (+2.19)	1.27
H3	62.65	2.48	63.43 (+0.78)	1.33

**Figure 8:**
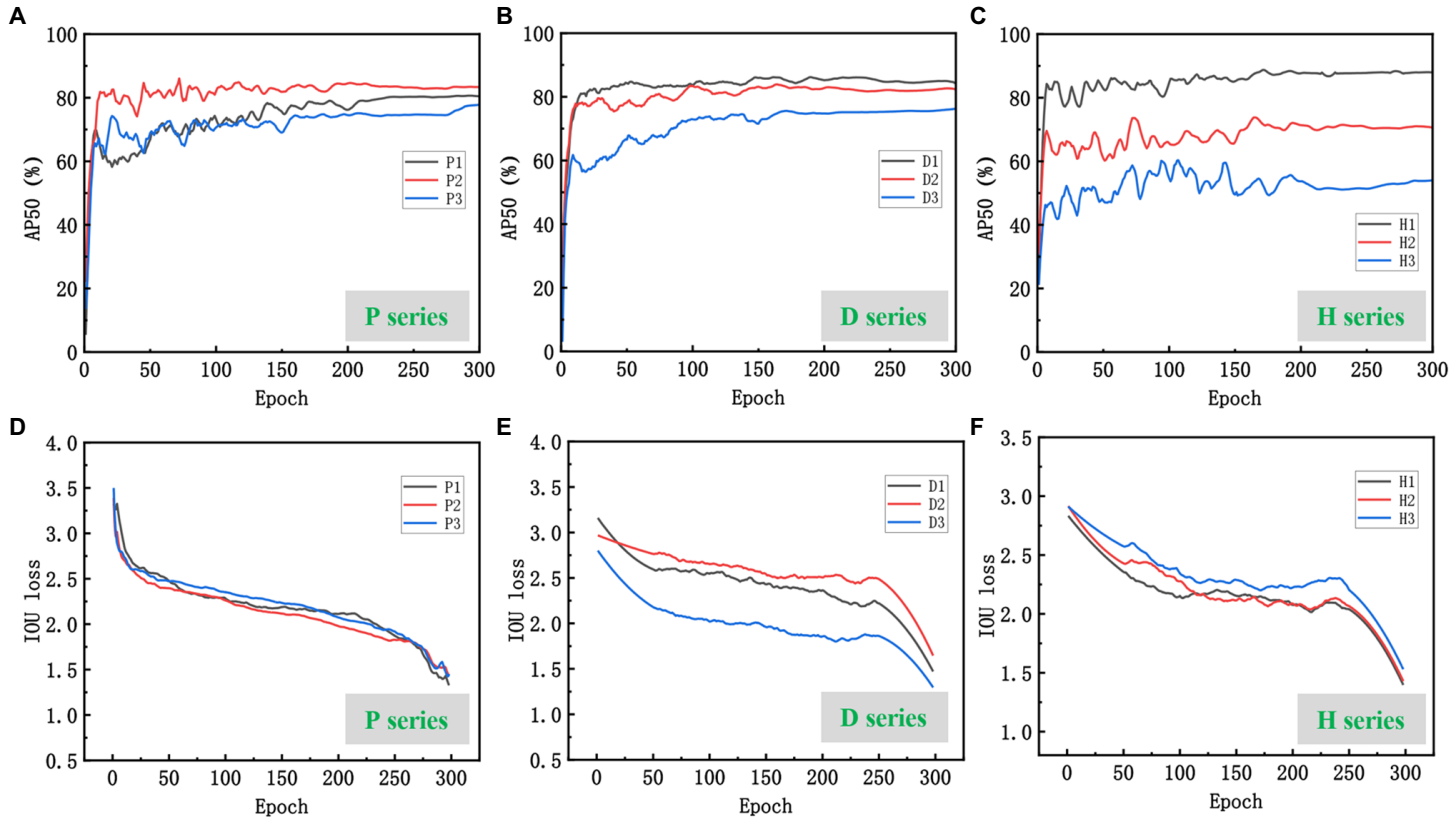
Performance of the improved model on the three series datasets: **(A–C)** are AP50 curves for the three series datasets; **(D–F)** are IOU loss curves for the three series datasets.

Scenario P: As shown in Figures 8A,D, the model performed best on the P2 dataset, with the AP50 quickly reaching the highest level and converging at the 180th epoch with a maximum AP of 88.03%. This is related to the strong contrast displayed by the wheat ears and leaves in the field during the P2 lactation period when the ears were grayish-white, differing significantly from the light green color presented by the leaves. Although slightly weaker than in the P1 flowering period, the P2 wheat-ear texture characteristics were significantly stronger than in the P3 maturity period (Figure 7A). The model showed a lower AP than P2 in the P1 flowering period, with a maximum AP of 80.70%, and the training curve was low at the beginning and then gradually increased, leveling off at 210 epochs, 7.33% lower than the best AP of P2. The color of wheat ears in the P1 flowering period was similar to that of the leaves, and the stacking of labeled boxes was slightly higher, indicating that the stacking of wheat ears in this period was more serious than in P2, which had an impact on the recognition of the model. The worst performance of the model was in the maturity period of P3, with the highest AP of 77.79%, 10.24% lower than the best AP of P2. The training curve started moderately, and the subsequent growth was slow, converging at 160 epochs and improving slightly, slowing down at the 270th epoch. The dataset statistics show that the wheat ears and leaves were green in this period. They are more similar, and the labeling frame stacking degree was up to 9.86%. The wheat ears are stacked to a high degree. These are the main reasons for the poor training accuracy of the model.

Scenario D: The higher the planting density, the denser the wheat ears in the same field of view of the camera, and the more severe the overlap. The model achieved 87.59% AP on the D1 dataset (Figures 8B,E), and the training curve converged early and fast. The D1 dataset had the lowest labeled frame overlap among all the datasets, at 2.98%, which is a more desirable dataset. Model training AP on the D2 dataset was 84.94%. With the increase of wheat planting density, the overlap between wheat ears and leaf shading gradually increased (Figure 7B), 2.65% compared with D1. The highest AP was only 76.23% in the D3 dataset with the highest test density, 11.36% lower than D1. Thus, the difficulty of wheat-ear recognition by UAV images increased sharply in wheat fields planted at high density.

Scenario H: The different flying heights of the UAVs affected the resolution of the images and the size of the target. And the size of the images intercepted in the three datasets in the H series experiment was 200 × 200 pixels (Figure 7C). The difference in the target object size in the H series dataset affects the model’s accuracy for wheat ear’s feature extraction and recognition. The larger target object, the more pronounced the target features extracted by the model and the higher the training and recognition accuracy. As shown in Figures 8C,F, the accuracy of the training curves of H1, H2, and H3 decreases in order, and the model performs best on the H1 dataset with the highest AP50 of 87.93% and the smallest fluctuation of the pre-training curve among the three periods and starts to converge first (about the 150th epoch); H2 has the second-highest training accuracy with the highest AP of 73.35% and starts to converge at the 180th epoch attachment. H3 has the worst effect, with the highest AP50 of only 63.43% and the most drastic fluctuations, there is a decline at 210 epochs. Then a slight upturn, and more wheat sheaves failed to be recognized by the model, as can be seen in the prediction effect graph.

Overall, combining the results of the three scenarios, the best UAV orthophoto recognition can be obtained from the wheat milk stage (P2), low planting density (D2), and low flight altitude (H1).

### Model Counting Accuracy Validation

**Figure 9:**
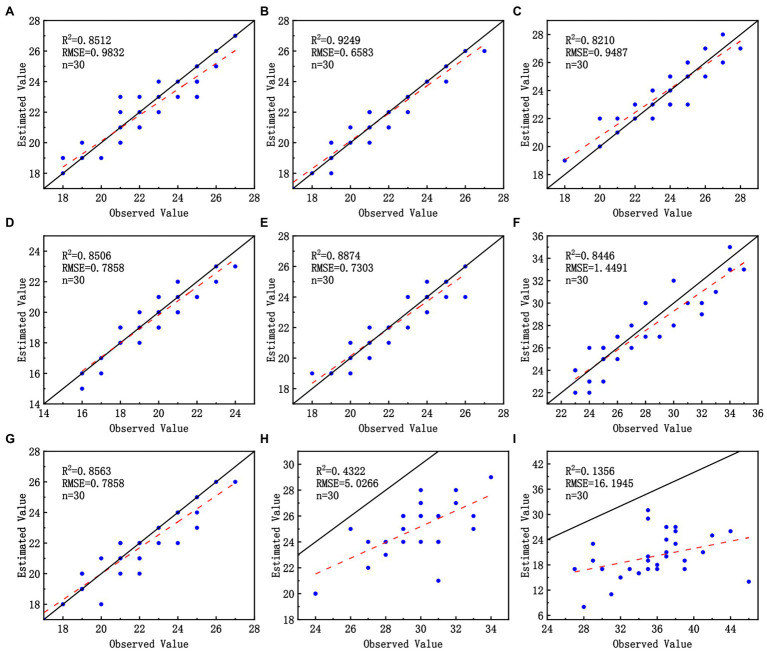
Performance of the improved model on three wheat scenes datasets: **(A–C)** correspond to P1, P2, and P3; **(D–F)** correspond to D1, D2, and D3; **(G–I)** correspond to H1, H2, and H3.

In each validation set, 30 images were randomly selected for manual recognition of wheat ears and counting. The results of manual observation of wheat-ear number and the recognition of wheat-ear number by the improved model were compared. The *R*^2^ and RMSE were calculated by linear fitting (Figure 9). Our model showed excellent wheat-ear recognition ability on all except H2 and H3 datasets, with *R*^2^ greater than 0.8446 and RMSE less than 1.4491. The best performance on the P2 dataset with an *R*^2^ of 0.9249 and an RMSE of 0.6583. And the model performed poorly on the H2 and H3 datasets due to the effect of the UAV flight height on the image GSD, which resulted in the wheat ears occupying too few pixels in the image; the features were difficult to be captured by the network, which also verifies that low GSD and small targets are difficult to identify with deep networks (Zhang et al., 2020).

## Discussion

### Analysis of Dataset Metrics

We counted and compared several metrics of the P, D, and H series datasets, including the average number of labeled boxes per figure ( $V_{lab}$ ), the stacking degree of labeled boxes ( $D_{lab}$ ), the average pixel of labeled boxes ( $V_{pix}$ ). $D_{lab}$ can approximate the degree of wheat stacking in the dataset. The equations of $V_{lab}$ , $D_{lab}$ , and $V_{pix}$ are as follows:


(9) $$V_{lab}=\frac{N_{lab}}{N_{img}}$$

(10) $$D_{lab}=\frac{lab_{i}\cap lab_{j}}{lab_{i}\cup lab_{j}}$$

(11) $$V_{pix}=\frac{\sum S_{pix}}{N_{lab}}$$

where $lab_{i}$ and $lab_{j}$ are any two annotation frames, and $S_{pix}$ is the sum of pixels occupied by all annotation frames.

Overall, as shown in Table 4, the number of images in each segmented scene dataset ranged from 255 to 395. The number of annotated boxes in each dataset was counted, with the least number of annotated boxes in the D1 dataset and the most in the P2 dataset. The average number of annotations reached a maximum of 36.0 and a minimum of 20.5.

**Table 4:** Statistical table of dataset indicators.

Dataset	P Series			D Series			H Series		
	P1	P2	P3	D1	D2	D3	H1	H2	H3
Average number of annotations (pcs)	23.1	24.6	23.9	20.5	24.2	30.4	24.5	30.0	36.0
>0.25 Stacking degree share (%)	5.22	3.97	6.68	2.68	5.95	7.47	3.42	3.15	3.67
Average annotation frame pixels (pixels)	984.5	1096.4	1164.3	930.4	1013.5	1087.1	1123.6	638.2	357.6

In the P series scenes, the average number of labeled frames per image in the P1 dataset was 23.1, with 5.22% of the frames having a stacking degree greater than 0.25 and an overall dark green color. Labeled frames per image in the P2 dataset were 24.6, with 3.97% of the frames having a stacking degree greater than 0.25 and an overall light green color. The average number of annotated frames per image in the P3 dataset was 23.9, with 6.86% of the annotated frames having a stacking degree greater than 0.25; they too were yellow overall. To make the network more successful in detecting wheat ears, it was necessary to expose the ears fully. Hence, they were visible in the images, with minimal leaf shading and overlap between ears. For different genotypes and environmental conditions, we observed wheat ears with different morphologies, sizes, and strain distributions. For example, in the case of Yangmai23, we observed that the wheat tended to bend during the seed filling stage, which increased the overlap between heads. However, in the stage between tasseling and flowering, some wheat spikes were not yet fully grown and were difficult to see. The wheat fertility stage affected the wheat plant morphology and thus the differences in the angle, overlap, and color of the wheat ears in the images taken by the UAV, and P2 was a period where better identification could be obtained. Therefore, we recommend acquiring images after flowering when the wheat ears are fully emerged and still upright.

Among the D series scenes, the average number of labeled frames per image in the low-density D1 scene was 20.5, and 2.68% of the labeled frames have a stacking degree greater than 0.25, with few labeled frames stacked. The average number of labeled frames per image in the D2 dataset was 24.2, and 5.95% of the labeled frames have a stacking degree greater than 0.25. The D3 dataset was the densest, and the overlap of labeled frames greater than 0.25 reached 7.47%. The density of wheat crop planting affected the recognition accuracy of the deep network; excessive density, serious stacking occurred, and the recognition effect decreased.

In the H series scenes, the average number of annotation frames per image increased with height, and the average size of wheat-ear annotation frames decreased with height, with 1123.6 pixels for H1, 638.2 pixels for H2, and 357.6 pixels for H3. Thus, the too-small size of wheat-ear annotation frames, i.e., the size of the pixels occupied by wheat ears, affected the recognition accuracy of the depth network.

### Constraint of Drone Flight Height

UAV orthophoto stitching needs to meet the synergy between parameters such as flight altitude, heading overlap rate, and side overlap rate. The UAV flight altitude is often set very low to obtain higher ground resolution (GSD) images. At too low a flight altitude, the strong wind from the UAV propeller blows the wheat plants about, making the wheat-ear tilt and swing and resulting in blurred photos and failed orthophoto stitching. This can prolong the mission time, and the data to be stored grow exponentially, requiring high UAV range and storage space. From the formula for GSD (Equation 12), it can be seen that the only condition that determines the GSD on a fixed focal length UAV is the UAV flight altitude, and too high a UAV flight altitude makes the GSD of the target object wheat ears too small for accurate identification. We tested the flight time, the number of photos, and data size required for different UAV flight heights and measured the GSD at different heights, as shown in Table 5. Taking Insprie2 with X5S and 45 mm fixed focal length lens as an example, setting the same heading overlap rate and side overlap rate, the lowest height that can achieve the route shooting task is 16 m, the flight time required for this task is 1.5 times as much as that of 20 m flight height task. A higher flight altitude can significantly reduce the number of photos and data size, and shorten the task execution time, while reducing the GSD.


(12) $$\mathrm{GSD}=\frac{H\times a}{f}$$

where *H* is the relative altitude, *f* is the focal length of the lens, and *a* is the image element size.

**Table 5:** Parameters corresponding to different flight altitudes of UAVs.

Flight altitude (m)	Heading overlap rate (%)	Sideways overlap rate (%)	GSD (cm)	Flight time (s)	Number of photos (pcs)	Data size (Mb)
15	78	80	-	-	-	-
20	78	80	0.3	569	241	2048.5
25	78	80	0.4	367	155	1317.3
30	78	80	0.5	263	110	935.7

The experiment found that the ground station software could not generate operational tasks at 15 m altitudes. Combining the completed three height scenarios GSD, and the results in sections “Ablation Experiments With Improved YOLOX-m Model, Performance of the Improved Model on Different Scenario Datasets, and Model Counting Accuracy Validation” shows that the UAV flight height has a significant impact on the model results, and it is necessary to ensure that the wheat is not affected by the wind while maximizing the shooting of a large target of small wheat ears under the limitations of UAV hardware. Using a 45 mm telephoto lens, a clear image (H1) was captured at a UAV altitude of 20 m, and a better model effect was achieved. Optimizing the UAV image acquisition method in balancing the relationship between flight altitude, mission time, and GSD, we found the best solution for the current hardware equipment conditions to improve the quality of UAV orthoimages, improve the characteristics of small wheat targets, and eliminate the imbalance of data by exhausting the construction of large-scale datasets and testing different sample distributions.

### Improving the Efficiency of UAV Recognition of Wheat Ears Using Larger Size Images

**Figure 10:**
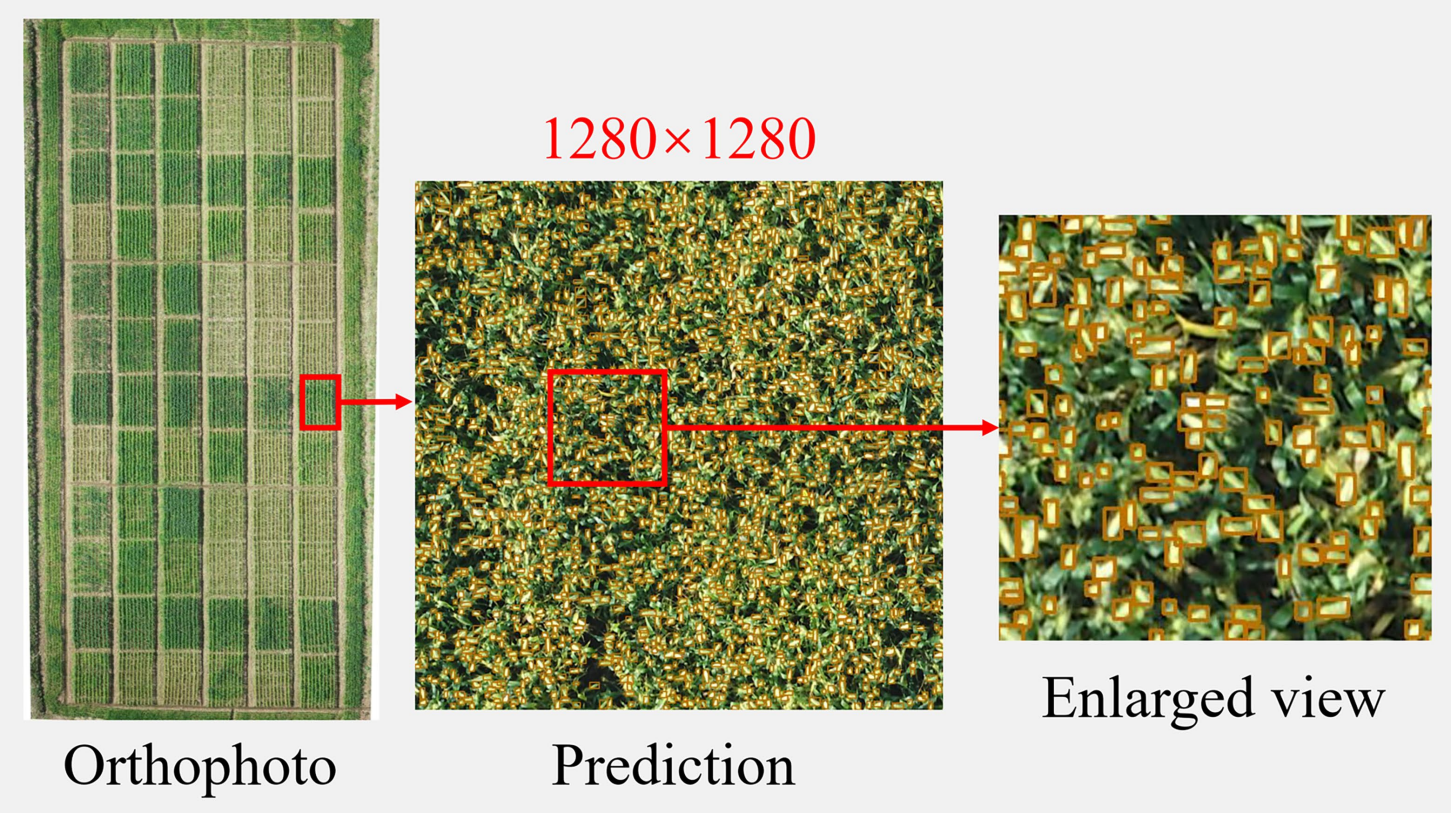
Orthophoto cropping recognition effect.

Finally, we tried to crop a random image of 1,280 × 1,280 pix on the UAV orthophoto and feed it into the improved model for prediction, as shown in Figure 10. The wheat ears could be recognized more accurately at this resolution, which indicates that the optimized network had a stronger perceptual ability and adapted to recognition at larger resolutions. The number of wheat ears in the manual statistics image was 1,689, and the network recognized 1,597, with an error of 5.45%. This method can reduce the segmentation of the UAV orthophoto processing into too many small images, which is very helpful in reducing the computation and image-processing times.

### Future Trends

Recent years have seen the research on UAV detection of wheat sheaves develop rapidly. However, it is still in the research stage, and more cost is needed to make this technology applicable to practical production. In addition, it has been found that thermal infrared images have better contrast than RGB images. The difference in temperature between wheat ears and other parts of the plant is used to segment the ears and delineate the color threshold for counting (Fernandez-Gallego et al., 2019). With the development of 3D technology and the popularity of 3D devices, 3D technology has also been applied to wheat-ear identification (Velumani, 2017; Ghahremani et al., 2021), using 3D laser point cloud segmentation technology to achieve wheat-ear identification, which provides another new idea for wheat-ear counting.

## Conclusion

This paper improved the YOLOX network by optimizing mosaics, adopting the BiFPN structure, and adding an attention mechanism. The ablation test showed that the change improved the network performance. Tests in three periods, at three densities, and for three height scenarios showed that our model had excellent wheat ear recognition on P1–P3, D1–D3, and H1 datasets, with *R*^2^ greater than 0.8446 and RMSE less than 1.4491, and the best performance on the P2 dataset with an *R*^2^ of 0.9249 and an RMSE of 0.6583. In comparison, H2 and H3 indicate that deep network recognition is difficult under the condition of low GSD. We suggest acquiring images after flowering when the wheat ears have fully emerged and are still upright. This sets up the best UAV flight plan with hardware devices to improve the quality of UAV orthoimages for the best training and recognition results.

## Data Availability Statement

The raw data supporting the conclusions of this article will be made available by the authors, without undue reservation.

## Author Contributions

YZ and LT: conceptualization. YZ: methodology, formal analysis, data curation, and writing—original draft preparation. JC: software. YT and JC: validation. SC: writing—review and editing and funding acquisition. LT: project administration. All authors contributed to the article and approved the submitted version.

## Funding

This research was financially supported by the National Natural Science Foundation of China (32172110, 32001465, and 31872852), the Key Research and Development Program (Modern Agriculture) of Jiangsu Province (BE2020319), and the Postgraduate Research Practice Innovation Program of Jiangsu Province (KYCX19_2107).

## Conflict of Interest

The authors declare that the research was conducted in the absence of any commercial or financial relationships that could be construed as a potential conflict of interest.

## Publisher’s Note

All claims expressed in this article are solely those of the authors and do not necessarily represent those of their affiliated organizations, or those of the publisher, the editors and the reviewers. Any product that may be evaluated in this article, or claim that may be made by its manufacturer, is not guaranteed or endorsed by the publisher.
